# Supplementary material for: Utility of an Untargeted Metabolomics Approach Using a 2D GC-GC-MS Platform to Distinguish Relapsing and Progressive Multiple Sclerosis
Source: Metabolites. 2024 Sep 11;14(9):493. doi: 10.3390/metabo14090493 (PMC11434588; doi:10.3390/metabo14090493)
Supplement: Supplementary file 1 [file metabolites-14-00493-s001.zip › Table S1.pdf]

Table 1: Differentially alttered metabolites in RRMS compared to HS

Compound Name	CAS	RRMS_t_stat	RRMS_t.test.p	RRMS_t.test.q
Methyl 11, 14-eicosadienoate (S)	61012-46-2	3.62804	0.00050	0.04510
11,14-Eicosadienoic acid		2.93677	0.00435	0.10732
2-Ethylhexanoic acid	149-57-5	3.51971	0.00096	0.05800
3-Aminoisobutyric acid		-2.35730	0.02112	0.27693
3-methyloctan-2-one		-2.17968	0.03227	0.28567
3-Phenoxy-1-propanol		2.16512	0.03326	0.28567
2,2-Dihydroxyacetic		-2.43307	0.01765	0.26527
a-D-Glucopyranose	492-61-5	3.08094	0.00284	0.10732
Erythrose	583-50-6	-2.86689	0.00535	0.10732
Succinic acid	110-15-6	-2.62638	0.01042	0.18790
a-Ketoisovaleric acid	759-05-7	2.25712	0.02663	0.28252
Ribose	50-69-1	3.69269	0.00041	0.04510
D-Glucuronic acid--lactone	32449-92-6	2.07498	0.04109	0.33686
D-Galactose	59-23-4	-2.31665	0.02303	0.27693
Formamide	75-12-7	2.18139	0.03200	0.28567
Margaric acid (C17)	506-12-7	2.88020	0.00507	0.10732
L-Tryptophan	73-22-3	-2.88146	0.00525	0.10732
L-Tyrosine	60-18-4	-2.99382	0.00364	0.10732
2-Hydroxypentanoic acid (S)		-2.33937	0.02173	0.27693
Ser-Leu		2.48064	0.01525	0.25003
