# Supplementary material for: Utility of an Untargeted Metabolomics Approach Using a 2D GC-GC-MS Platform to Distinguish Relapsing and Progressive Multiple Sclerosis
Source: Metabolites. 2024 Sep 11;14(9):493. doi: 10.3390/metabo14090493 (PMC11434588; doi:10.3390/metabo14090493)
Supplement: Supplementary file 1 [file metabolites-14-00493-s001.zip › Table S2.pdf]

Table 2: Differentially alttered metabolites in PPMS compared to HS

Compound Name	CAS	PPMS_t.test.t	PPMS_t.test.p	PPMS_t.test.q
d-Mannose	3458-28-4	-3.73059	0.00040	0.02218
Margaric acid (C17)	506-12-7	-3.67706	0.00044	0.02218
11,14-Eicosadienoic acid		-3.65283	0.00048	0.02218
Methyl 11, 14-eicosadienoate (S)	61012-46-2	-2.79928	0.00692	0.13397
2,3-Dihydroxybutanoic acid	5057-93-2	-2.73406	0.00822	0.13397
Glycerol	56-81-5	-2.58773	0.01165	0.13397
1-Monopalmitin		-2.53230	0.01339	0.13397
D-(+)-Xylose	58-86-6	-2.52315	0.01378	0.13397
a-ketoisocaproic acid	816-66-0	-2.41092	0.01833	0.13706
Pentadecanoic acid (C15)	1002-84-2	-2.37339	0.02074	0.13706
Dimethyl tartarate [R-(R*,R*)]-		-2.37139	0.02063	0.13706
Erythrose	583-50-6	-2.36158	0.02081	0.13706
2-Hexyldecanol		-2.16849	0.03347	0.20131
trans-Oleic acid	112-79-8	-2.11532	0.03819	0.22012
Myristic acid		-2.07169	0.04214	0.22419
2-Hydroxybutyric acid		-2.04563	0.04491	0.23008
Pipecolic acid		2.07853	0.04113	0.22419
L-(-)-Arabitol	7643-75-6	2.32845	0.02496	0.15694
L-Asparagine	70-47-3	2.44714	0.01711	0.13706
L-Valine	72-18-4	2.48659	0.01549	0.13397
2,5,7,7-tetramethylcyclohepta-1,3,5-triene		2.49566	0.01498	0.13397
2-Hydroxypentanoic acid (S)		2.50149	0.01474	0.13397
10-Undecenoic acid	112-38-9	2.58105	0.01175	0.13397
L-Tyrosine	60-18-4	2.66105	0.00965	0.13397
9,12-Octadecadiynoic acid	2012-14-8	3.04238	0.00345	0.07944
D(-)-Tagatose	87-81-0	3.34473	0.00129	0.04456
