# Supplementary material for: Utility of an Untargeted Metabolomics Approach Using a 2D GC-GC-MS Platform to Distinguish Relapsing and Progressive Multiple Sclerosis
Source: Metabolites. 2024 Sep 11;14(9):493. doi: 10.3390/metabo14090493 (PMC11434588; doi:10.3390/metabo14090493)
Supplement: Supplementary file 1 [file metabolites-14-00493-s001.zip › Table S3.pdf]

Table 3: Differentially alttered metabolites in MS (RRMS and PPMS) compared to HS

Compound Name	CAS	RRMS_t_stat	RRMS_t.test.p	RRMS_t.test.q	PPMS_t.test.t	PPMS_t.test.p	PPMS_t.test.q
11,14-Eicosadienoic acid		2.93677	0.00435	0.10732	-3.65283	0.00048	0.02218
2-Hydroxypentanoic acid (S)		-2.33937	0.02173	0.27693	2.50149	0.01474	0.13397
Erythrose	583-50-6	-2.86689	0.00535	0.10732	-2.36158	0.02081	0.13706
L-Tyrosine	60-18-4	-2.99382	0.00364	0.10732	2.66105	0.00965	0.13397
Margaric acid (C17)	506-12-7	2.88020	0.00507	0.10732	-3.67706	0.00044	0.02218
Methyl 11, 14-eicosadienoate (S)	61012-46-2	3.62804	0.00050	0.04510	-2.79928	0.00692	0.13397
